# bak deletion stimulates gastric epithelial proliferation and enhances *Helicobacter felis*-induced gastric atrophy and dysplasia in mice

**DOI:** 10.1152/ajpgi.00404.2014

**Published:** 2015-07-09

**Authors:** C. A. Duckworth, A. A. Abuderman, M. D. Burkitt, J. M. Williams, L. A. O'Reilly, D. M. Pritchard

**Affiliations:** ^1^Department of Gastroenterology, Institute of Translational Medicine, University of Liverpool, Liverpool, United Kingdom;; ^2^The Walter and Eliza Hall Institute of Medical Research, Parkville, Victoria, Australia; and; ^3^Department of Medical Biology, The University of Melbourne, Parkville, Victoria, Australia

**Keywords:** bcl-2 family, *Helicobacter felis*, gastric cancer

## Abstract

*Helicobacter* infection causes a chronic superficial gastritis that in some cases progresses via atrophic gastritis to adenocarcinoma. Proapoptotic bak has been shown to regulate radiation-induced apoptosis in the stomach and colon and also susceptibility to colorectal carcinogenesis in vivo. Therefore we investigated the gastric mucosal pathology following *H. felis* infection in *bak*-null mice at 6 or 48 wk postinfection. Primary gastric gland culture from *bak*-null mice was also used to assess the effects of *bak* deletion on IFN-γ-, TNF-α-, or IL-1β-induced apoptosis. *bak*-null gastric corpus glands were longer, had increased epithelial Ki-67 expression, and contained fewer parietal and enteroendocrine cells compared with the wild type (wt). In wt mice, bak was expressed at the luminal surface of gastric corpus glands, and this increased 2 wk post-*H. felis* infection. Apoptotic cell numbers were decreased in *bak*-null corpus 6 and 48 wk following infection and in primary gland cultures following cytokine administration. Increased gastric epithelial Ki-67 labeling index was observed in C57BL/6 mice after *H. felis* infection, whereas no such increase was detected in *bak*-null mice. More severe gastric atrophy was observed in *bak*-null compared with C57BL/6 mice 6 and 48 wk postinfection, and 76% of *bak*-null compared with 25% of C57BL/6 mice showed evidence of gastric dysplasia following long-term infection. Collectively, *bak* therefore regulates gastric epithelial cell apoptosis, proliferation, differentiation, mucosal thickness, and susceptibility to gastric atrophy and dysplasia following *H. felis* infection.

bcl-2 proteins are key regulators of apoptosis. The roles of bcl-2 family members have been previously investigated in the gastrointestinal tract, and several of these proapoptotic proteins are important regulators of spontaneous and induced apoptosis in both the intestine and stomach (9, 19, 24, 28, 29, 31). bak is a proapoptotic member of this family of proteins and is expressed in the normal stomach epithelium, with strong immunoreactivity being observed in gastric pits and parietal cells. However, no bak expression is observed in mucous neck cells or chief cells (11, 17). Gastric tumors also express reduced levels of bak compared with normal gastric mucosae (16). *bak* gene mutations have been reported in human gastrointestinal cancers, suggesting that a disturbance in bak-mediated apoptosis may contribute to the pathogenesis of gastric cancer (15, 35). Furthermore, bak has previously been shown to regulate susceptibility toward radiation-induced apoptosis in both the colon (9) and stomach (31). Gastric antral and corpus epithelial apoptosis was increased in *bcl-2*-null and decreased in *bax*-null and *bak*-null mice following 12 Gy γ-irradiation (31). Understanding the mechanisms involved in regulating gastric epithelial apoptosis and factors that may confer differences in susceptibility to gastric injury may therefore provide insights into the development of gastric cancer.

*Helicobacter pylori* infection is the best-characterized risk factor for the development of gastric adenocarcinoma in humans (13). Intestinal-type distal gastric cancer develops following the preneoplastic stages of atrophic gastritis, intestinal metaplasia, and dysplasia (5); however, the precise etiology *of Helicobacter*-induced gastric cancer remains to be determined. Wild-type mice infected with *H. pylori* do not usually develop gastric cancer, whereas C57BL/6 animals infected with *H. felis* develop gastric neoplasia 15 mo following infection; this is therefore considered to be a good model of *H. pylori* infection in humans (10). Defects in apoptotic regulation have been shown to play a role in carcinogenesis in multiple organ systems, including the gastrointestinal tract (9, 24, 30, 40, 41), and apoptotic processes in the stomach are known to be altered during the early phases of *Helicobacter* infection (6, 36, 39). Elevated numbers of gastric cells undergoing apoptosis have previously been reported as a consequence of *H. pylori* infection in humans and *H. felis* infection in mice (6, 8, 12, 20, 25), and several bcl-2 family members have shown differential expression patterns following infection by *H. pylori* (11). Both INS-GAS mice that overexpress human amidated gastrin and mice rendered hypergastrinaemic by infusion of gastrin also have perturbed expression of bcl-2 family members (7). These mice are more susceptible than their wild-type counterparts to *H. felis*-induced apoptosis, develop more severe gastric atrophy, and show a faster progression to gastric cancer (7, 32, 38). The prevention of Fas-mediated apoptosis, however, has been shown to accelerate the progression and increase the invasiveness of gastric cancer in mice (3). The induction of apoptosis by IFN-γ along with beclin-1 induced autophagy, and reduction in progenitor cell expansion has also resulted in the prevention of *H. felis*-induced neoplasia (37). These findings suggest differential roles for the intrinsic and extrinsic apoptotic pathways during gastric carcinogenesis.

Understanding the ways in which various genes regulate apoptosis following *H. felis* infection may therefore aid in understanding the pathogenesis of gastric cancer. In the colon, bak expression plays a role in both regulating cellular differentiation and in the maintenance of colonic crypt length (9). To date, the effects of *bak* deletion on gastric differentiation and proliferation have not been determined. We have therefore investigated whether bak is important in maintaining homeostasis in the untreated murine gastric epithelium and also whether this protein regulates the consequences of *H. felis* infection in mice.

## MATERIALS AND METHODS

### 

#### Animals.

B6.129-*Bak1*^*tm1Thsn*^/J (*Bak*-null) mice (19) were purchased from Jackson Labs (stock no. 004183; Bar Harbor, ME). *bak*-null mice were maintained on the C57BL/6J (C57BL/6) genetic background. *bax* wild-type and homozygously null mice on a mixed genetic background came from stock as described by Knudson et al. (14) and were generated from heterozygous mating at the University of Liverpool. Comparisons were made between wild-type C57BL/6J (Charles River Laboratories, Kent, UK) and *bak*-null and between *bax*-null and *bax*-wt mice with a minimum of 5 mice/experimental group. Mice were housed in a conventional animal house facility with a 12:12-h light-dark cycle, fed a commercially available diet, and given water ad libitum. All experiments were conducted with the United Kingdom Home Office and University of Liverpool ethics committee approval.

#### Animal treatments.

Male and female C57BL/6 and *bak*-null mice were orally gavaged at 6 wk of age with ∼10^8^ *H. felis* (ATCC 49179) in 0.5 ml on 3 days over the course of 1 wk. Infected mice and untreated control mice maintained in parallel were killed by cervical dislocation at various time points following infection from 2 to 48 wk. Untreated C57BL/6 and *bak*-null mice were used aged 10–12 wk for ex vivo gastric gland cultures.

#### Tissue preparation.

Following death, ligatures were tied around the distal esophagus and proximal duodenum, and the stomach was inflated with 4% formal saline. Following overnight fixation, tissue was processed and embedded in paraffin wax, and 3- to 5-μm sections of antrum and corpus were stained with hematoxylin and eosin, alcian blue-PAS, or prepared for immunohistochemistry.

#### Histological scoring methods.

Twenty antral and 40 corpus hemiglands per mouse were assessed on a cell positional basis starting from cell position 1 at the gland base and ending at the gastric lumen for each of the stained or immunolabeled tissue sections, as previously described in the stomach (31) and in more detail in the small intestine and colon (23, 28). Data are presented as mean labeling index or as labeling index against cell position. The ratio of proliferation to apoptosis was determined by dividing the number of Ki-67-positive cells per hemigland by the number of caspase-3-positive cells per hemigland for each mouse. Degree of colonization by *H. felis* was determined from hematoxylin (H)- and eosin (E)-stained circumferences of antrum and was scored as follows: 0, no *Helicobacter*-like organisms observed; 1, light colonization of <10 *Helicobacter*-like organisms observed in ≥5 crypts/antral circumference; and 2, heavy colonization of ≥10 *Helicobacter*-like organisms observed in ≥5 glands/antral circumference. Visual-analog scoring from H and E and alcian blue/PAS histological sections was carried out by a European College of Veterinary Pathologists certified pathologist (Williams) and was used to determine the severity of the histological parameters of inflammation, mucous metaplasia, epithelial defects, and dysplasia using previously defined criteria (2, 33).

#### Immunohistochemistry.

Immunohistochemistry was performed on formalin-fixed paraffin-embedded antral and corpus sections. Antibodies used were rabbit anti-*bak* (Millipore, Hampshire, UK), rabbit antiactive caspase-3 (R & D Systems, Oxfordshire, UK), rabbit antichromogranin A (Abcam, Cambridge, UK), rabbit anti-H^+^-K^+^-ATPase (Calbiochem, Nottingham, UK), and rat anti-Ki-67 (Dako, Ely, UK). Immunostaining for bak, active caspase-3, chromogranin A, and Ki-67 was performed as previously described (9, 21). Briefly, antigen retrieval was performed by heat retrieval in 10 mmol/l tricarboxylic acid buffer (pH 6), and the primary antibody was applied at 1:600 for bak, 1:750 for active caspase-3, 1:1,000 for chromogranin A, and 1:20 for Ki-67 overnight at 4°C. A goat antirabbit or rabbit antirat biotinylated secondary antibody (Dako) was used at a dilution of 1:200, and an ABC (Vector Laboratories, Peterborough, UK) amplification step was performed before detection by 3-3′-diaminobenzidine tetrahydrochloride. Immunohistochemistry for H^+^-K^+^-ATPase did not require heat-mediated antigen retrieval, instead, sections were incubated with 1% Triton X-100 for 30 min before the addition of primary antibody at 1:1,000.

#### Murine primary gastric gland preparation and culture.

Murine primary gastric glands were isolated from five male and five female 10- to 12-wk-old C57BL/6 and *bak*-null stomachs using a method described in detail previously (26). Briefly, a ligature was tied around the proximal duodenum to isolate the stomach, and the forestomach was discarded. The stomach was inverted and inflated with collagenase A (0.5 mg/ml) and washed for 10 min in 1 mM dithiothreitol in Hank's balanced salt solution (HBSS; Sigma, Gillingham, UK) before being washed in HBSS three times and placed back to digest in collagenase A (0.35 mg/ml) solution at 37°C in a shaking water bath at 100 revolutions/min in an atmosphere of 95% O_2_ and 5% CO_2_ until gastric glands were dislodged in solution. Glands were allowed to settle for 45 min before being plated in 12-well tissue culture plates on glass cover slips (Appleton Woods, Selly Oak, UK) that contained 1.5 ml/well DMEM-Ham's F-12 mix (Sigma), 10% FCS (Invitrogen, Paisley, UK), 1.25% l-glutamine (Sigma), and 1% antibiotic/antimycotic mixture (Sigma). Following digestion and plating, glands were maintained at 37°C in a humidified environment containing 5% CO_2_ for 24 h. Media was changed to fresh complete media for a further 24 h, and then 48 h following initial plating, TNF-α (150 ng/ml; PeproTech, London, UK), IL-1β (150 ng/ml; PeproTech), or IFN-γ (150 ng/ml; PeproTech) was added to cultures in fresh media for 48 h before fixation in 2% formaldehyde for 30 min followed by three washes in PBS.

#### Gastric gland primary culture immunofluorescence and scoring.

Immunofluorescence was carried out for active caspase-3. Nonspecific binding was blocked for 1 h using 10% normal goat serum, 1% BSA, and 0.1% Triton X-100 and then incubated with rabbit antiactive caspase-3 primary antibody (1:900; R & D Systems) overnight at 4°C. A goat antirabbit FITC-conjugated secondary antibody was applied for 40 min at room temperature in the dark (1:200; Stratech Scientific, Newmarket, UK). Cover slips were mounted on glass slides with antifade mounting medium containing DAPI (Vector Laboratories) and assessed on a fluorescent microscope with a ×40 objective (Olympus, Southend-on-Sea, UK). Ten discrete glands each containing at least 20 cells were scored per animal from each treatment group. The total number of DAPI-stained nuclei and the total number of active caspase-3-positive cells were assessed per gland and presented as mean gland size and percentage of cells positive for active caspase-3. Data were normalized to untreated glands of each genotype.

#### Statistical analysis.

A two-way ANOVA followed by Holm-Sidak post hoc analysis was performed on all normally distributed data unless otherwise indicated in the text. Results are expressed as means ± SE. A modified median test was used to determine differences at individual cell positions. Differences were deemed as significant when *P* < 0.05 by ANOVA and when cell positional distributions were different at three or more consecutive cell positions (27). Nonparametric data were assessed by Kruskal-Wallis ANOVA on ranks followed by Dunn's post hoc analysis.

## RESULTS

### 

#### bak-null mice show increased corpus gland length and increased epithelial cell proliferation.

**Fig. 1.**
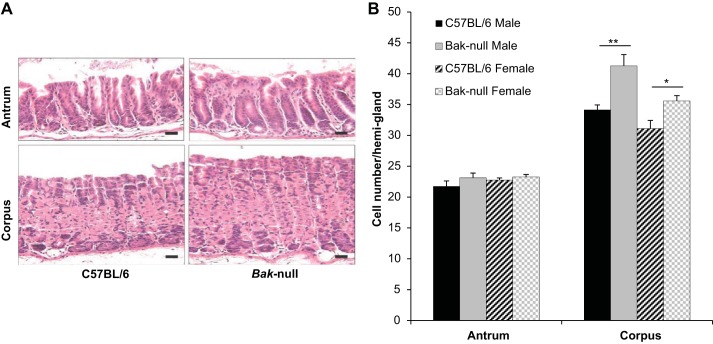
*A*: hematoxylin (H) and eosin (E) photomicrographs showing gastric antrum (*top*) and corpus (*bottom*) from C57BL/6 (*left*) and *bak*-null (*right*) mice. *B*: cell no./hemigland from C57BL/6 (black) and *bak*-null (gray) male (solid) and female (hatched) gastric antrum and corpus. **P < 0.01 and *P < 0.05 by ANOVA followed by Holm-Sidak post hoc test.

Because *bak*-null mice showed colonic crypt hyperplasia but did not show any differences in small intestinal crypt or villus length (9), we determined whether the gastric glands of these mice displayed an altered proliferative response. The physiological structure of gastric antral glands is very different from that of corpus glands; therefore, these regions were assessed separately. The gastric corpus glands of *bak*-null were significantly longer than those of their C57BL/6 wild-type counterparts in both male and female mice; however, antral glands showed no significant difference in length between sex or strain (Fig. 1, *A* and *B*). bak has previously shown functional redundancy with bax, another proapoptotic bcl-2 family member (19). We therefore quantified gland length in *bax*-null compared with *bax*-wt mice and showed no significant difference in gastric corpus gland length (data not shown). However, corpus gland length of three *bak/bax*-null mice (22) was increased to a similar extent to that shown in *bak*-null mice at 43 ± 3 cells/hemigland, suggesting that bak was solely responsible for the observed perturbations in gastric gland length.

**Fig. 2.**
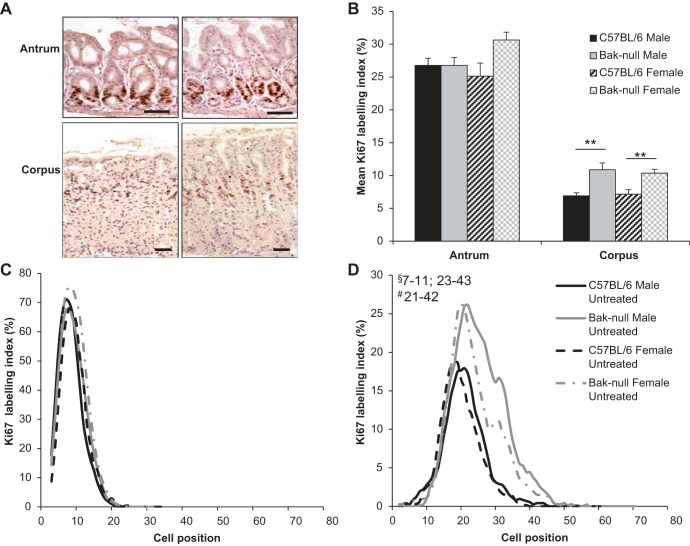
*A*: Ki-67 immunohistochemistry showing antrum (*top*) and corpus (*bottom*) from C57BL/6 (*left*) and *bak*-null (*right*) mice. *B*: Ki-67 labeling index in C57BL/6 (black) and *bak*-null (gray) male (solid) and female (hatched) gastric antrum and corpus. *C* and *D*: cell positional distribution of Ki-67-positive epithelial cells from C57BL/6 (black) and *bak*-null (gray) male (solid) and female (dotted) gastric antrum (*C*) and gastric corpus (*D*). **P < 0.01 by ANOVA and Holm-Sidak post hoc analysis and significantly different cell positions by modified median test indicated between genotypes on graphs. §, Male; #, female.

*bak*-null male and female gastric corpus glands contained significantly more Ki-67-positive cells per gland than those in C57BL/6 mice (Fig. 2, *A* and *B*). Peak Ki-67 expression was observed at similar cell positions from the gland base in male and female C57BL/6 and *bak*-null mice; however, *bak*-null male and female mice showed significant changes in the distribution of Ki-67-positive cells, with a greater abundance of Ki-67-positive cells being located further along the gland axis toward the gastric lumen compared with C57BL/6 mice (Fig. 2*D*). However, no difference in Ki-67 labeling was observed between sex or genotype in the gastric antrum (Fig. 2, *A* and *B*), and no significant difference was observed in the distribution of Ki-67-positive cells in the gastric antrum of any sex or strain of mouse (Fig. 2*C*).

#### Spontaneous apoptosis in the gastric corpus is not affected by bak deletion.

A reduction in the amount of spontaneous apoptosis was observed in the colon following *bak* deletion (9). Baseline expression of active caspase-3 was very low in the stomach (corpus: C57BL/6, 0.05 ± 0.02; *bak*-null, 0.05 ± 0.01; antrum: C57BL/6, 0.04 ± 0.02; *bak*-null, 0.01 ± 0.01 cells/hemigland), and therefore any differences in expression are likely to have been below the limit of detection. We therefore found no significant difference in baseline amount of active caspase-3 expression between the two strains of mice in either males or females (data presented later as part of Fig. 7*A*).

#### Gastric epithelial differentiation is perturbed by bak deletion.

**Fig. 3.**
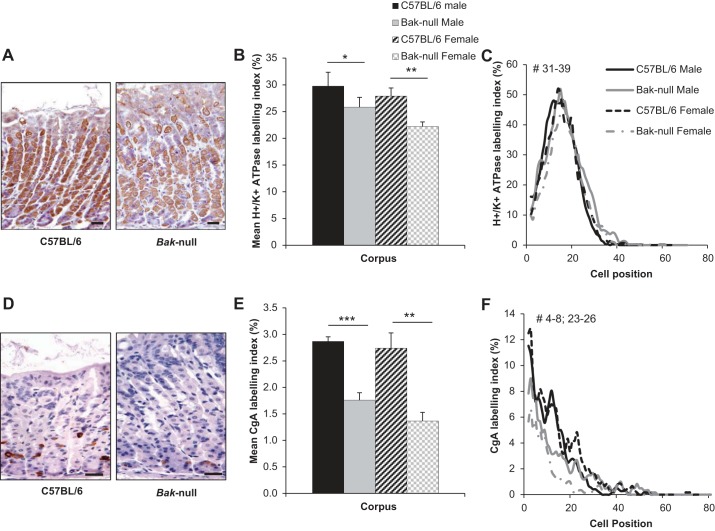
*A*: H^+^-K^+^-ATPase immunohistochemically stained gastric corpus from C57BL/6 (*left*) and *bak*-null (*right*) mice. *B*: H^+^-K^+^-ATPase labeling index in gastric corpus from C57BL/6 (black) and *bak*-null (gray) male (solid) and female (hatched) mice. *C*: cell positional distribution of H^+^-K^+^-ATPase-positive epithelial cells from C57BL/6 (black) and *bak*-null (gray) male (solid) and female (dotted) gastric corpus. *D*: chromogranin A immunohistochemically stained gastric corpus from C57BL/6 (*left*) and *bak*-null (*right*) mice. *E*: chromogranin A labeling index in gastric corpus from C57BL/6 (black) and *bak*-null (gray) male (solid) and female (hatched) mice. *F*: cell positional distribution of chromogranin A-positive epithelial cells from C57BL/6 (black) and *bak*-null (gray) male (solid) and female (dotted) gastric corpus. ***P < 0.001, **P < 0.01, and *P < 0.05 by ANOVA and Holm-Sidak post hoc analysis. ^#^Significantly different cell positions by modified median test indicated between C57BL/6 and *bak*-null female mice.

*bak*-null mice show altered colonic cellular differentiation with goblet cell hyperplasia and enteroendocrine cell hypoplasia (9). Because the gastric corpus glands of *bak*-null mice were longer and had elevated proliferation indexes (Figs. 1 and 2) compared with their wild-type counterparts, we also assessed the regulation of secretory cell lineage differentiation in the gastric corpus epithelium of these mice. A significant reduction in the percentage of parietal cells was observed in both male and female *bak*-null gastric corpus compared with wild-type controls (Fig. 3, *A* and *B*). Additionally there was a significant change in parietal cell distribution between male *bak*-null and C57BL/6 animals between cell positions 31–39, which is likely due to the observed increase in gland length (Fig. 3*C*). To determine whether there was any alteration in the cellular abundance of enteroendocrine lineages, we assessed the localization of chromogranin A. Male and female *bak*-null mice showed a significant reduction in the percentage of chromogranin A-positive epithelial cells (Fig. 3*E*) with significant distribution differences being observed in both male and female mice (Fig. 3*F*). Alcian blue/PAS-stained slides were also assessed for any changes in mucin expression or localization within the *bak-*null corpus; however, no differences in alcian blue/PAS distribution or abundance of acid mucosubstances (blue staining) and neutral polysaccharides (magenta staining) were observed compared with C57BL/6 male or female mice (data not shown). Immunohistochemistry was also carried out for the gastric mucin Muc5AC, and again no change in percentage abundance or distribution was observed between *bak*-null and C57BL/6 mice (data not shown).

#### bak expression is increased following H. felis infection.

**Fig. 4.**
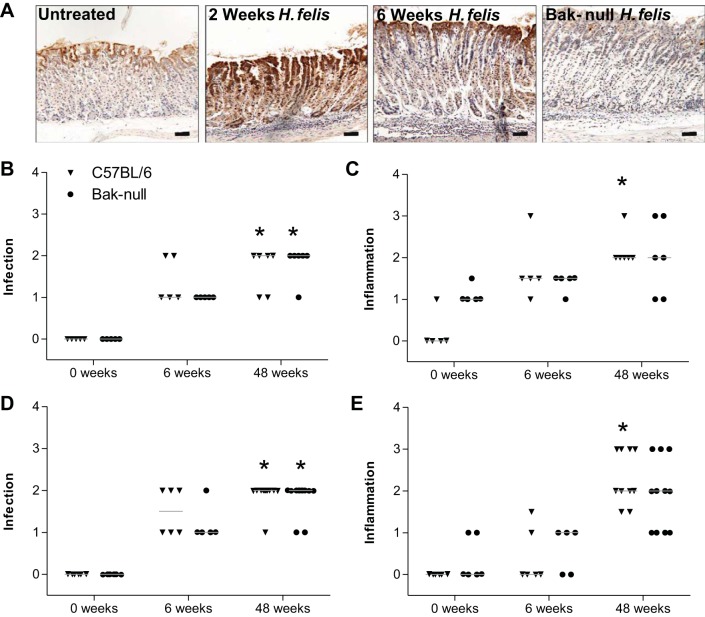
*A*: immunohistochemical staining for bak in untreated and 2 and 6 wk *H. felis*-infected C57BL/6 mice and *H. felis*-infected *bak*-null mouse corpus. Visual analog scoring of C57BL/6 (triangle) and *bak*-null (circle) mice for infection [female (*B*) and male (*D*)] and inflammation [female (*C*) and male (*E*)]. *P < 0.05, significantly different compared with control untreated group of the same genotype by Kruskal-Wallis ANOVA with Dunn's post hoc analysis.

Lamina propria expression of bak has been shown to gradually increase over 20 wk of infection by *H. pylori* in mice (4, 11), and expression of bcl-2 family members is also altered in hypergastrinaemic mice, which may be a significant factor responsible for their increased susceptibility to *H. felis*-induced gastric carcinogenesis (7, 38). bak expression was therefore evaluated in C57BL/6 wild-type mice following infection with *H. felis* and was found to be increased as early as 2 wk post-*H. felis* infection, returning to normal levels between 6 and 20 wk postinfection (Fig. 4*A*).

#### bak protects against gastric corpus inflammation.

Female C57BL/6 mice are more susceptible to *H. felis*-induced gastric pathology than male mice on the same genetic background (6). Male and female *bak*-null and C57BL/6 mice were therefore used in *H. felis* infection experiments and assessed separately. *H. felis* colonization indexes were determined in the gastric antrum of *bak*-null mice 6 or 48 wk following infection. No differences in *H. felis* colonization were observed between *bak*-null and C57BL/6 antrum at either 6 or 48 wk postinfection in either male or female mice (Fig. 4, *B* and *D*). As anticipated, C57BL/6 mice showed a significantly increased severity of inflammation 48 wk following *H. felis* infection compared with uninfected control mice. This significant increase in inflammation grading was, however, not observed in the *bak*-null group, since untreated *bak*-null gastric corpus displayed a slightly higher grade of baseline inflammation (median score of 1) compared with C57BL/6 (median score of 0). However, no significant difference in the inflammation grading between genotypes was observed at either 6 or 48 wk post-*H. felis* infection in male or female animals (Fig. 4, *C* and *E*).

#### bak-null mice show attenuated hyperplastic changes following H. felis infection.

**Fig. 5.**
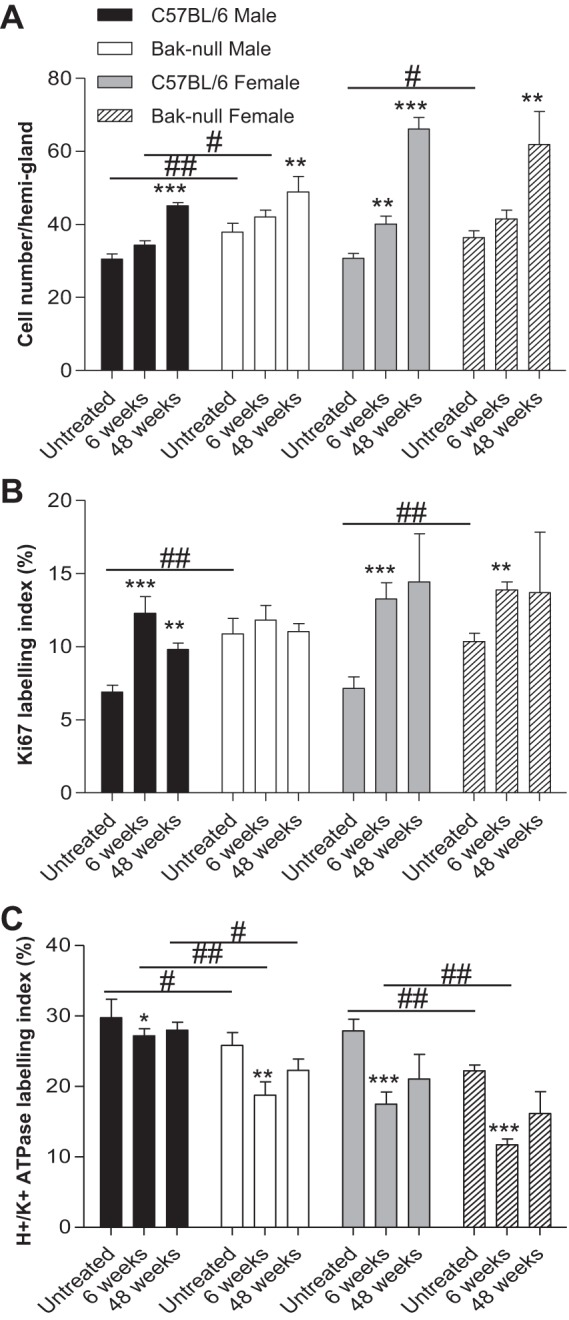
Cell number per hemigland (*A*), mean Ki-67 labeling index (*B*), and mean H^+^-K^+^-ATPase labeling index (*C*) for C57BL/6 and *bak*-null male and female gastric corpus either untreated or 6 or 48 wk following infection with *H. felis*. ***P < 0.001, **P < 0.01, and *P < 0.05 between infected and untreated of the same sex and genotype. ^##^*P* < 0.01 and ^#^*P* < 0.05 between genotypes of the same sex and administered with the same treatment by 2-way ANOVA followed by Holm-Sidak post hoc analysis.

We proceeded to further characterize the responses of *bak*-null mice to *H. felis* infection. *H. felis* infection has previously been shown to induce gastric corpus gland hyperplasia, with an expanded proliferative zone being observed 8 wk postinfection in female C57BL/6 mice (6). Corpus gland length in C57BL/6 mice was therefore assessed and was significantly increased 6 wk post *H. felis* infection as expected; however, *bak*-null mice showed no such increase (Fig. 5*A*). Forty eight weeks after *H. felis* infection, significant hyperplastic changes were evident in all mice compared with uninfected control mice of the same sex and genotype (Fig. 5*A*). Because untreated *bak*-null mice have longer gastric corpus glands than C57BL/6 mice, the increase in gland length 6 wk following infection was blunted compared with C57BL/6 gastric corpus glands, and a further increase beyond that of C57BL/6 mice was not observed (Fig. 5*A*).

Proliferation index was assessed by Ki-67 immunohistochemistry and is shown as the percentage of total cells per gland, since the observed hyperplastic changes induced by *H. felis* infection, particularly at 48 wk postinfection, were so immense. C57BL/6 and *bak*-null gastric corpus showed a significant increase in Ki-67 labeling 6 wk post-*H. felis* infection compared with untreated controls of the same genotype; however, this increase was less pronounced in *bak*-null mice compared with C57BL/6 (Fig. 5*B*). This phenomenon was also apparent when assessed on a total Ki-67-positive cell per hemigland basis (untreated: C57BL/6 2.3 ± 0.34, *bak*-null 3.7 ± 0.26; 6 wk infected: C57BL/6 5.47 ± 0.32, *bak*-null 5.46 ± 0.23; 48 wk infected: C57BL/6 11.52 ± 3.8, *bak*-null 9.1 ± 3.6). Percentage expression of Ki-67 in both genotypes did not significantly change between 6 and 48 wk following *H. felis* infection (Fig. 5*B*). Whereas Ki-67-labeled cells were found in greater proportion at cell positions nearer to the lumen of gastric corpus glands in untreated *bak*-null mice (Fig. 2*D*), this change in distribution between genotypes was not apparent following *H. felis* infection (data not shown).

#### bak-null mice are more susceptible to developing gastric atrophy following H. felis infection.

It is well documented that infection with *H. felis* results in parietal cell atrophy (8). We therefore assessed the percentage of H^+^-K^+^-ATPase-labeled parietal cells 6 and 48 wk following *H. felis* infection in *bak*-null and C57BL/6 gastric corpus glands. Significant parietal cell loss was observed in C57BL/6 and *bak-*null gastric corpus 6 wk following *H. felis* infection compared with untreated control mice of the same sex and genotype (Fig. 5*C*). The degree of parietal cell loss was, however, more marked in *bak*-null mice than C57BL/6 mice, and significantly less parietal cells were observed in male and female *H. felis*-infected *bak*-null compared with *H. felis*-infected C57BL/6 gastric corpus (Fig. 5*C*), suggesting that they are more susceptible to developing gastric corpus atrophy.

#### Bak deletion enhances dysplastic lesions following H. felis infection.

**Fig. 6.**
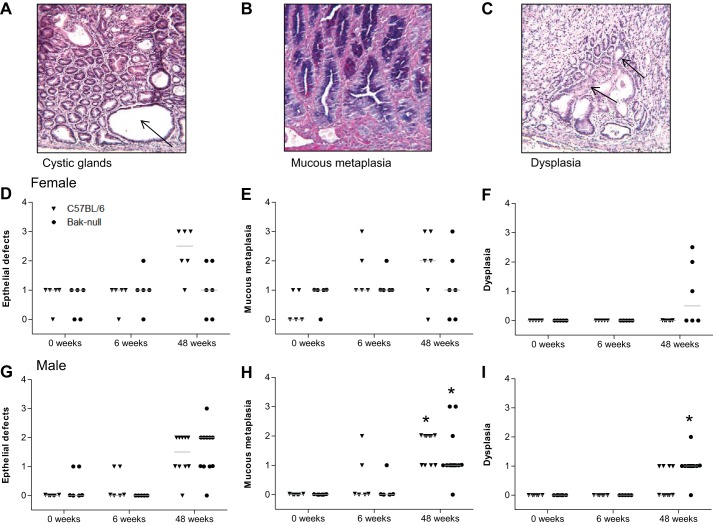
H&E image of cystic glands (*A*), alcian blue/PAS image of mucous metaplasia (*B*), and H&E image of dysplasia (*C*). Visual analog scoring of C57BL/6 (triangle) and *bak*-null (circle) gastric corpus for epithelial defects [female (*D*) and male (*G*)], mucous metaplasia [female (*E*) and male (*H*)], and dysplasia [female (*F*) and male (*I*)]. *P < 0.05, significantly different compared with control untreated group of the same sex and genotype by Kruskal-Wallis ANOVA with Dunn's post hoc analysis.

Epithelial defects (where gastric glands are dilated ± attenuation of surface epithelium; Fig. 6*A*), mucous metaplasia (where cells exhibit foamy change, with increased expression of acidic and neutral mucins within glands; Fig. 6*B*), and dysplasia (where glands exhibit irregular structure, in which there is increased epithelial crowding and loss of columnar polarity; Fig. 6*C*) were also quantified in infected C57BL/6 and *bak*-null gastric corpuses. Increases in mucous metaplasia and epithelial defects were observed over the time course of infection in the gastric corpus of male and female C57BL/6 mice. Similar amounts of epithelial defects (Fig. 6, *D* and *G*) and mucous metaplasia (Fig. 6, *E* and *H*) were observed in infected *bak*-null mice, but the changes relative to baseline were not statistically significant. By 48 wk post-*H. felis* infection, however, 3 out of 6 female and 10 out of 11 male *bak*-null mice displayed evidence of dysplasia, which included irregular glands that exhibited loss of orientation, distortion, and branching and crowding of mildly pleomorphic epithelial cells, with markedly hyperchromatic nuclei, and increased nuclear-to-cytoplasmic ratio. In contrast, mild dysplasia was only observed in 4 out of 10 male C57BL/6 mice 48 wk postinfection (Fig. 6, *F* and *I*).

#### bak-null female mice show a reduced apoptotic response to H. felis infection.

**Fig. 7.**
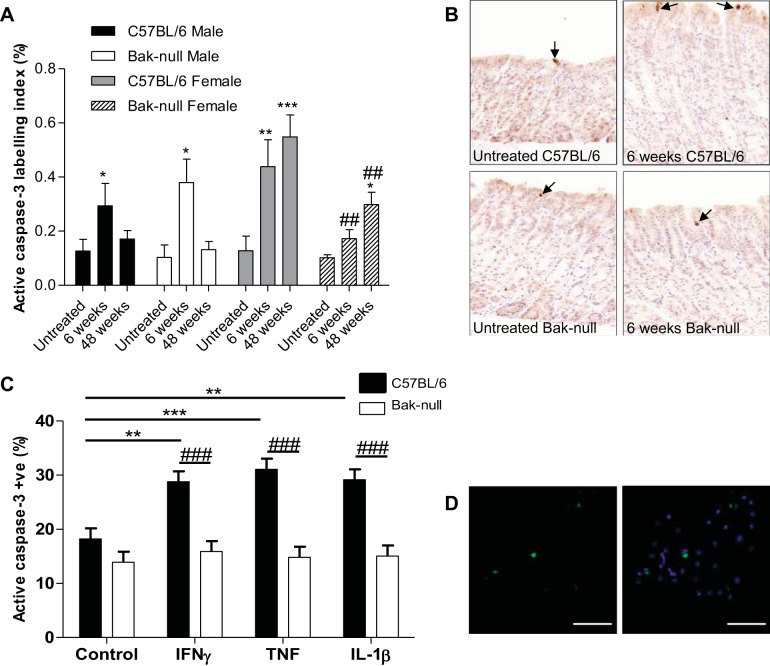
*A*: mean active caspase-3 labeling index for C57BL/6 and *bak*-null male and female gastric corpus either untreated or following 6 or 48 wk of infection with *H. felis*. *B*: active caspase-3-immunostained sections from untreated C57BL/6 and *bak*-null gastric corpus (*left*) and 6 wk following *H. felis* infection (*right*). Apoptotic bodies are indicated by black arrows. *C*: percentage active caspase-3 labeling from primary gastric gland cultures from C57BL/6 (black) and *bak*-null (white) mice treated with proinflammatory cytokines IFN-γ, TNF, or IL-1β. *D*: isolated C57BL/6 gastric gland in 2-dimensional in vitro culture stained with active caspase-3 (green, *left*) and overlayed with DAPI-stained nuclei (blue, *right*). ***P < 0.001, **P < 0.01, and *P < 0.05 between infected and untreated of the same genotype. ^###^*P* < 0.001 and ^##^*P* < 0.01 between genotypes administered with the same treatment by 2-way ANOVA followed by Holm-Sidak post hoc analysis.

*H. felis* infection has previously been shown to increase gastric epithelial cell apoptosis (6, 8, 12, 20, 25). We therefore assessed the apoptotic index using active caspase-3 immunohistochemistry as a readout in *bak*-null mice 6 and 48 wk following infection with *H. felis* to determine whether any alteration in apoptotic response contributed to the observed increase in dysplasia. Mean apoptotic index in C57BL/6 mice was increased following infection at the 6-wk time point in both males and females and remained elevated 48 wk following infection in females, but returned to baseline amounts in males. However, this elevation in the proportion of apoptotic cells was not observed in female *bak*-null mice at 6 wk, which remained blunted compared with C57BL/6 mice at 48 wk following infection. A significant increase in active caspase-3 labeling index 48 wk following *H. felis* infection was observed in female *bak*-null gastric corpus compared with untreated *bak*-null corpus glands; however, the magnitude of this increase was again lower than that observed in C57BL/6 mice (Fig. 7*A*). A significant reduction in apoptotic index was observed between C57BL/6 and *bak*-null female mice at both 6- and 48-wk time points following *H. felis* infection (Fig. 7*A*), but this reduction was not observed in males. In summary, the proliferation-to-apoptosis ratio within gastric corpus glands was therefore elevated at each time point in *bak*-null female mice (untreated: 91 ± 22; 6 wk infected: 70 ± 17; 48 wk infected: 38 ± 13) compared with C57BL/6 mice (untreated: 55 ± 19; 6 wk infected: 25 ± 5; 48 wk infected: 20 ± 6), reaching statistical significance (*P* < 0.05) by *t*-test for the 6-wk-infected group only.

#### bak-null gastric glands have an attenuated response to cytokine-induced apoptosis.

Because *bak*-null mice showed a reduced gastric corpus epithelial cell apoptotic index following infection compared with C57BL/6 mice (Fig. 7, *A* and *B*) but displayed no differences in inflammatory infiltrate at 6 and 48 wk following *H. felis* infection (Fig. 4, *C* and *E*) and because *H. felis*-induced pathology is thought to be mediated by an increase in Th1 cytokines, we assessed the direct effects of three cytokines known to be expressed in abundance following *H. felis* infection (10) on isolated gastric glands from C57BL/6 and *bak*-null mice. There was no significant difference in the percentage of active caspase-3-positive cells in unstimulated *bak*-null primary gastric gland cultures compared with C57BL/6; however, when treated with IFN-γ, TNF, or IL-1β, a significant increase in active caspase-3 labeling was observed in C57BL/6 gastric glands, whereas no such increase above the baseline unstimulated amount of active caspase-3 staining was observed in *bak*-null gastric glands (Fig. 7, *C* and *D*).

## DISCUSSION

We have demonstrated that the proapoptotic bcl-2 family member bak plays an important role in regulating gastric epithelial cell homeostasis. bak regulates gastric corpus gland length and both proliferative and apoptotic responses in the stomach in a similar way to that which we have previously described in colonic crypts (9). Alterations in the relative proportions of several differentiated cell types were also observed in the stomach as previously shown in the colon (9). In both tissues, *bak*-null mice showed a similar reduction in endocrine cell number. In the colonic mucosa, this phenotype was associated with an increase in *Gfi1*, and a decrease in *Neuro D1* and *Pax6* expression (9). In the gastric corpus, bak deletion also resulted in a decrease in the proportion of H^+^-K^+^-ATPase-expressing parietal cells, another secretory cell lineage. We determined that these phenomena were exclusive to bak deletion, since no increase in gland length was observed in *bax*-null gastric corpus and a similar increase in corpus gland length was observed in *bak/bax*-null mice. However, the observed differences in tissue homeostasis in *bak*-null gastric corpus were somewhat less marked than in the colon. We therefore also sought to determine whether bak expression modulated gastric gland homeostasis following *H. felis*-induced injury, since we have previously shown that apoptosis in both gastric corpus and antral glands of *bak*-null mice was reduced following 12 Gy γ-radiation (31).

bak expression has previously been observed in the surface and pit cells of the normal murine gastric epithelium and to increase following *H. pylori* infection in this species (11). Similarly, gastric biopsies from *H. pylori*-infected patients show cytoplasmic bak expression toward the luminal surface of gastric glands, whereas biopsies from control patients show little to no protein expression in this region (4). bak is the only multi-BH domain proapoptotic protein to be highly expressed in the gastric corpus (11) and is therefore likely to be an important component of the gastric corpus apoptotic machinery. Increased bak expression and apoptosis is also seen in human gastric cancer cell lines (e.g., AGS) following *H. pylori* infection (4).

*H. felis* infection of mice is a well-characterized model of human distal gastric cancer development. In this study, we showed that bak expression was also elevated in epithelial cells that are located at the luminal surface and pits of C57BL/6 mouse gastric glands 2 wk following *H. felis* infection. Although this location of bak expression has previously been described, the importance of its increased expression following *H. felis* infection has not previously been determined.

We observed a greater number of gastric corpus epithelial cells and Ki-67-positive epithelial cells in untreated *bak*-null mice, suggesting that the gastric corpus glands of *bak*-null mice are longer than those of C57BL/6 wild-type mice due to an enhanced rate of proliferation. It is interesting that the deletion of a gene that is known for its role in the regulation of apoptosis is also able to influence the capacity of gastric glands to proliferate. This has also been demonstrated following siRNA knockdown of bak in IEC-18 (rat ileal epithelial cell 18) cells and in the colon in vivo where altered expression of cyclin D1, p16, and p27 was found to be associated with this proliferative response (9, 18). There is evidence to suggest that the expansion of the proliferative compartment in gastrointestinal epithelia is a risk factor for gastrointestinal cancers (1). This may therefore implicate bak deletion as a risk factor for the development of gastric cancer. Baseline amounts of apoptosis in the gastric epithelium showed no significant difference between genotypes, and additionally an increased proliferation-to-apoptosis ratio was observed in *bak*-null mice, suggesting that the mechanism by which bak stimulates gastric gland hyperplasia is not entirely through enhanced longevity as a result of a reduction in apoptosis but through apoptosis-independent mechanisms. Spontaneous apoptotic events in the stomach are relatively rare (31), and, without an apoptotic stimulus, differences between genotypes may therefore fall below the limit of detection. We have previously described a reduction in intercrypt table apoptosis in the colonic epithelia of *bak*-null mice and have suggested that reduced cell death in the colonic epithelium along with an increased abundance of mitotic figures in the stem cell region may be the mechanism responsible for the observed colonic crypt hyperplasia in these mice (9). Basal levels of apoptosis are, however, higher in the colon compared with the stomach. These phenomena were not apparent in the gastric antrum where gland length, Ki-67 expression, and active caspase-3 expression were unaltered in *bak*-null animals, suggesting that bak deletion has little influence over gastric mucosal architecture in this region.

*H. felis* infection of mice increases the number of apoptotic bodies that are observed in epithelial cells at the luminal surface of both gastric corpus and antrum, causes enhanced parietal cell atrophy in the gastric corpus, and results in gastric corpus gland hyperplasia (6, 8, 34). Given that bak expression was elevated over the time course of infection by *H. felis* in the murine gastric mucosa within 2 wk and returned to baseline levels of expression at 6–20 wk following infection, and that there was an expanded proliferative compartment in the gastric corpus of untreated *bak*-null mice, we determined how *bak*-null mice responded to both short (6 wk)- and long (48 wk)-term infections with *H. felis*. Despite elevated baseline expression of the proliferative marker Ki-67, there was no difference in Ki-67 labeling index between infected C57BL/6 and *bak*-null mice at both the 6- and 48-wk postinfection time points, suggesting that the rate of increase in the number of proliferative cells was slower in *bak*-null mice following infection by *H. felis*. It is currently unclear why the proliferative zone of *bak*-null gastric glands does not continue to expand at the same rate as wild-type C57BL/6 glands following short- and long-term infection by *H. felis*; however, there may be a bak-independent mechanism that controls the maximal number of cells with the capacity to proliferate in gastric corpus glands.

In contrast, a greater degree of parietal cell atrophy was observed in *bak*-null mice at both the 6- and 48-wk time points following *H. felis* infection compared with C57BL/6 mice. This suggests that bak may play a role in regulating parietal cell numbers in response to infection. Interestingly, despite the increase in parietal cell atrophy observed in *bak*-null mice, we were unable to detect a difference in severity of inflammatory infiltrate or degree of *H. felis* infection compared with C57BL/6 wild-type animals. This suggests that the deletion of bak in immune cells may not be directly responsible for the observed proliferative, apoptotic, and atrophic changes that we observed following *H. felis* infection and that these changes are likely to be a consequence of bak deletion in the epithelium itself. Seventy six percent of 48-wk *H. felis*-infected *bak*-null mice developed gastric corpus dysplasia, whereas only 25% of control C57BL/6 animals that were infected at the same time developed dysplastic glands. The reduced capacity for epithelial cells to undergo apoptosis in the gastric corpus and heightened proliferation-to-apoptosis ratio in gastric epithelial cells may therefore result in cells that have an increased susceptibility to neoplastic progression following DNA damage. This is in contrast to Cui et al. (7) who reported that increased amounts of apoptosis were associated with gastric carcinogenesis in INS-GAS mice. The INS-GAS mice used in this study were, however, on the FVB/N genetic background, and males demonstrated a greater susceptibility to *H. felis*-induced pathology compared with females, showing a sex difference between mouse strains. The mechanisms of apoptosis induction between the two models may also be different, suggesting a complex link between apoptosis and gastric susceptibility to carcinogenesis.

Quantification of active caspase-3-positive epithelial cells in the gastric corpus revealed a significantly lower number in *bak*-null compared with C57BL/6 female mice at both 6 and 48 wk following *H. felis* infection. Increased apoptotic cells were only observed at the 6-wk time point in male mice of both genotypes, which is consistent with the reduced *H. felis*-induced pathology that is observed in this sex. Potential bak activators that may be responsible for this reduction in apoptosis following infection by *H. felis* were assessed in primary gastric gland cultures from *bak*-null and C57BL/6 mice. Cytokines that are known regulators of apoptosis in *H. felis*-induced gastritis, namely IFN-γ, TNF, and IL-1β, did not induce apoptosis beyond baseline levels in isolated *bak*-null gastric glands; however, they produced an ∼1.6-fold increase in the number of active caspase-3-positive cells in C57BL/6 gland cultures 48 h following treatment. Cultured gland sizes were similar from both strains of mouse, suggesting that this is not a result of *bak*-null glands proliferating at a greater rate. These results indicate that the reduction in apoptotic cells observed following *H. felis* infection in vivo may be as a consequence of reduced sensitivity to these cytokines.

In conclusion, our studies demonstrate that bak deletion in mice is associated with increased susceptibility toward developing gastric cancer. In addition to its known role in promoting apoptosis, bak also regulates the size of the proliferative zone and differentiation pathways within the gastric epithelium, and also modulates the apoptotic response of this tissue following disease-causing stimuli such as infection by *Helicobacter* species. We have also demonstrated a role for bak in the prevention of gastric atrophy and dysplasia during the course of *H. felis* infection in mice. Perturbed regulation of both proliferation and apoptosis, severity of gastric atrophy, and dysplasia are implicated in the susceptibility to gastric cancer. Further studies are now warranted to assess the mechanisms by which bak suppresses these protumorigenic effects, to determine novel approaches for anticancer therapy.

## GRANTS

C. A. Duckworth was funded by a North West Cancer Research grant to D. M. Pritchard. A. A. Abuderman was funded by the Saudi Arabian government. M. D. Burkitt was funded by a Wellcome Trust Clinical Research Training Fellowship, a Wellcome Trust/University of Liverpool Institutional Strategic Support Fund grant, and a CORE/BSG development grant. J. M. Williams was funded by the Centre for Integrative Mammalian Biology, University of Liverpool. L. A. O'Reilly was funded by a project grant from the Cancer Australia funding partner, Cancer Council New South Wales no. 1047672, a National Health and Medical Research Council infrastructure grant, Independent Research Institutes Infrastructure Support Scheme Grant no. 361646, and the Victorian State Government (OIS grant).

## DISCLOSURES

No conflicts of interest, financial or otherwise, are declared by the authors.

## AUTHOR CONTRIBUTIONS

C.A.D., A.A.A., M.D.B., and L.A.O. performed experiments; C.A.D., A.A.A., M.D.B., and J.M.W. analyzed data; C.A.D., A.A.A., M.D.B., J.M.W., and D.M.P. interpreted results of experiments; C.A.D. prepared figures; C.A.D. and D.M.P. drafted manuscript; C.A.D., A.A.A., M.D.B., J.M.W., L.A.O., and D.M.P. edited and revised manuscript; C.A.D., A.A.A., M.D.B., J.M.W., L.A.O., and D.M.P. approved final version of manuscript; D.M.P. conception and design of research.
